# Allylamines, Benzylamines, and Fungal Cell Permeability: A Review of Mechanistic Effects and Usefulness against Fungal Pathogens

**DOI:** 10.3390/membranes12121171

**Published:** 2022-11-22

**Authors:** Dalal Hammoudi Halat, Samar Younes, Nisreen Mourad, Mohamad Rahal

**Affiliations:** 1 Department of Pharmaceutical Sciences, School of Pharmacy, Lebanese International University, Bekaa 146404, Lebanon; 2 Department of Biomedical Sciences, School of Pharmacy, Lebanese International University, Bekaa 146404, Lebanon

**Keywords:** naftifine, terbinafine, butenafine, ergosterol, cell membrane, fungi, dermatophytes

## Abstract

Allylamines, naftifine and terbinafine, and the benzylamine, butenafine, are antifungal agents with activity on the fungal cell membrane. These synthetic compounds specifically inhibit squalene epoxidase, a key enzyme in fungal sterol biosynthesis. This results in a deficiency in ergosterol, a major fungal membrane sterol that regulates membrane fluidity, biogenesis, and functions, and whose damage results in increased membrane permeability and leakage of cellular components, ultimately leading to fungal cell death. With the fungal cell membrane being predominantly made up of lipids including sterols, these lipids have a vital role in the pathogenesis of fungal infections and the identification of improved therapies. This review will focus on the fungal cell membrane structure, activity of allylamines and benzylamines, and the mechanistic damage they cause to the membrane. Furthermore, pharmaceutical preparations and clinical uses of these drugs, mainly in dermatophyte infections, will be reviewed.

## 1. Introduction to Drugs with Activity on Fungal Membranes—Summary/History of Allylamines and Benzylamines

**Figure 1:**
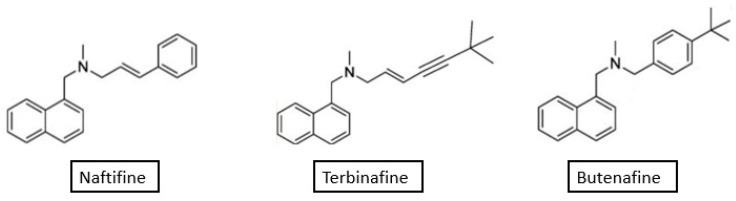
Chemical structures of naftifine, terbinafine, and butenafine. Structures were retrieved from data deposited in or computed by PubChem (https://pubchem.ncbi.nlm.nih.gov, accessed on 10 November 2022).

Fungal infections are a global health problem associated with high morbidity and mortality [1,2]. They can range in severity from superficial infections that affect the skin or nails, to severe invasive or disseminated infections that are life-threatening [3,4]. The advocacy group Global Action Fund for Fungal Infections (GAFFI) estimates that over 300 million people of all ages suffer from a serious fungal infection each year globally. Notably, around 1.35 million of these people are estimated to die from their fungal infections [5]. Fungi are eukaryotic organisms that are classified as a separate kingdom because of their unique cell walls that contain glucans and chitin, therefore, their eradication necessitates different strategies than those required for treating bacterial infections [6]. Available antifungal agents may be categorized according to their molecular targets [7]. They may affect membrane permeability, synthesis of membrane and cell wall components, synthesis of nucleic acids, and microtubule/mitotic spindle function [8]. Ergosterol is the predominant component of the fungal cell membrane but not a component of mammalian cell membrane [9]. Therefore, antifungal agents such as azoles, polyenes, and allylamines/benzylamines/thiocarbamates, which exert their antifungal activities through inhibition of synthesis of or direct interaction with ergosterol, can effectively suppress fungal cell growth with minimal effects on mammalian cells [10,11,12]. Allylamines, naftifine and terbinafine, and the benzylamine, butenafine, are examples of antifungal agents that interfere with the fungal cell membrane function. The first derivative of the allylamines class was naftifine, which was discovered at the Sandoz Research Institute in Vienna, Austria, in 1974 [13]. It was proven to be highly effective in vivo and in vitro against a significant number of fungi that are pathogenic to humans [13] and has been marketed as a topical antifungal since 1985 [14]. As for terbinafine, it is a synthetic allylamine derivative that was discovered in 1991 and was approved in the United States for topical use in 1992 and as an oral antifungal agent in 1998 [15]. Butenafine is the first and only agent in the benzylamine class of antifungals [16]. It is a synthetic topical antifungal that is structurally and pharmacologically related to allylamines with a butylbenzyl group in the place of the allylamine group [17,18]. In this review, the structure of fungal cell membrane and its lipids, and the mechanism of action of allylamines and benzylamines in light of affecting membrane permeability are discussed. In addition, available pharmaceutical preparations and clinical uses of these drugs, mainly in dermatophyte infections, are presented. The structures of the three antifungal compounds addressed in this review are shown below in Figure 1.

## 2. The Structure and Lipids of the Fungal Membrane

**Figure 2:**
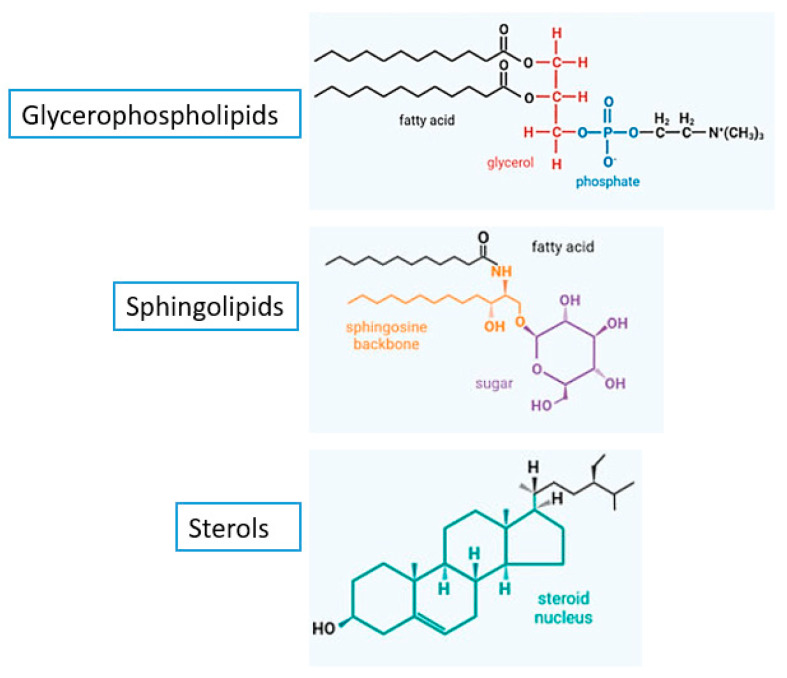
Representative structures of lipids present in fungal cell membranes [29]. Glycerophospholipids possess both polar glycerol and phosphate group and nonpolar hydrocarbon. Sphingolipids have a sphingosine backbone and an attached sugar. Sterols have a steroidal nucleus composed of four tightly fused carbon rings with a hydroxyl group attached to the first ring.

The fungal plasma membrane along with its cell wall provide both mechanical strength for the cell to withstand turgor pressure, hydrodynamic pressure higher than atmospheric pressure and applying a force on the cell wall, and to protect against toxic agents including antifungals [19]. Mammals lack the main constituents of fungal membranes, which makes it possible to specifically target them with compounds that do not interfere with the human cell metabolism [19]. Lipids are important biomolecules for the survival of all cells. They determine generic physical properties of the membrane such as its thickness, surface charge, fluidity, and intrinsic curvature [20,21]. Glycerophospholipids, sphingolipids, and sterols (Figure 2), three different classes of lipids, are abundant in the cell membranes of fungi [22]. Glycerophospholipids serve as essential structural elements of cell membranes and are crucial for fungal growth development [23]. They constitute about 55–75% of the lipids in fungal membranes and their amphipathic nature drives the formation of the membranes’ lipid bilayer structure that forms the basic skeleton of the cell membrane [19]. Glycerophospholipids consist of a glycerol-3-phosphate with two fatty acyl chains that are mostly unsaturated [24,25]. Based on their head groups, glycerophospholipids can be further subdivided into phosphatidylcholine (PC), phosphatidylethanolamine (PE), phosphatidylinositol (PI), phosphatidylserine (PS), and so forth. The amounts and types of phospholipids found in the plasma membrane of eukaryotic cells vary [26]. For instance, PC, PE, PI, and PS make up the major phospholipids in total cell extracts of the yeast *Saccharomyces cerevisiae* [27]. Compared to human cell membranes, fungal membranes are more negative because of the different ratio of neutral and anionic phospholipids [28]. Due to these electrostatic differences, cationic antifungal peptides prefer the membranes of pathogenic yeast to the host membranes as their target [28].

The fungal plasma membrane is also enriched with sphingolipids; a group of lipids that are ubiquitous and crucial for the membrane’s structure and function [30,31]. They constitute about 7–16% of the fungal membrane lipids [19,28]. These lipids are composed of a backbone of sphingosine containing long-chain aliphatic amino alcohols named sphingoid bases [22,32]. The synthesis of the sphingoid bases (sphingosine, dihydrosphingosine, and ceramides) from nonsphingolipid precursors occurs on the cytoplasmic face of the endoplasmic reticulum and is catalyzed by serine palmitoyltransferase [32]. Sphingolipids and sterols join to form microdomains in the fungal membrane known as lipid rafts, which are essential for growth, development of cell polarity, formation of hyphae, and pathogenicity [33,34]. In the plasma membranes of fungi, sphingolipid-enriched domains coexist with a wide array of membrane compartments that differ markedly from the mammalian lipid rafts in being larger, more temporally stable microdomains with a better-defined localization [30]. Moreover, sphingolipids control cellular processes including apoptosis and senescence by acting as signaling molecules [35]. Fungal sphingolipids are structurally different from their mammalian counterparts, demonstrating the possibility for sphingolipids to be used as novel targets for selective antifungal medications [36].

In their turn, sterols, also known as steroid alcohols, are vital components of fungal cell membranes that are important for their growth and viability [37]. They form around 30–40% of membrane lipids [38]. They are amphipathic lipids with rigid and compact ring structures [22] that play a variety of functions including the regulation of the membrane’s fluidity, the control of membrane-bound enzymes’ activity, and the maintenance of the membrane’s permeability [39]. Fungal cell membrane typically contains ergosterol as the major sterol that functions to maintain its integrity in the same capacity as cholesterol, the main component of mammalian cell membranes [37]. Ergosterol is described as a “fungal hormone” that can promote growth and proliferation [40]. It has been recently demonstrated to be an immunoactive lipid that causes host cell’s pyroptosis, a form of necrotic and inflammatory programmed cell death [41,42]. Additionally, ergosterol has been lately shown to be vital for fungal mitochondrial DNA maintenance, whereby ergosterol biosynthesis inhibition has led to mitochondrial DNA loss in *S. cerevisiae* [43,44]. This highlights the important role that ergosterol plays in stress adaptation during fermentation in fungi [45], as the fungal ability to tolerate stress was closely related to the levels of ergosterol [46].Therefore, ergosterol homeostasis is crucial for fungal cells, including controlling the transcription of genes which encode ergosterol biosynthetic enzymes and proteins needed for sterol processing and uptake [39,47].

The synthesis of ergosterol occurs in the endoplasmic reticulum through the sequential activity of many enzymes that mutually cooperate for regulating ergosterol content [45,48]. This pathway is complex and consumes a significant amount of energy [45]. Although fungi and humans share a strikingly similar process for sterols biosynthesis, both pathways differ in their later stages, rendering two structurally distinct molecules: ergosterol and cholesterol, which fulfill the cellular and membrane requirements [37]. Some of the genes involved in the early steps of ergosterol synthesis are classified as essential genes and those include ERG1, ERG7, ERG9, ERG11, ERG24, ERG25, ERG26, and ERG27, whereas others are considered as non-essential ones [45,49]. For instance, squalene synthase, an enzyme that catalyzes the biosynthesis of squalene and a key ergosterol precursor, is encoded by ERG9. Furthermore, another two essential enzymes in the ergosterol synthesis pathway are squalene epoxidase and lanosterol synthase, which are encoded by ERG1 and ERG7, respectively. Additionally, lanosterol 14-alpha demethylase is encoded by ERG11 and functions as an enzyme of the fungal cytochrome P450 family [49]. Hence, the majority of clinically available antifungals target ergosterol due to its specific biosynthesis pathway, distinctive structural properties, and critical functions [42,48].

Additionally, the fungal membrane is made up of a huge variety and number of proteins that serve a wide range of functions. The ATPase family, secondary transport proteins, and proteins involved in signal transduction, cell wall synthesis, and cytoskeleton production are among the major protein families that can be found in fungal plasma membrane [50].

## 3. Mechanism of Action of Allylamines and Benzylamines

Allylamines, such as naftifine and terbinafine, are a class of antifungals acting as ergosterol biosynthesis inhibitors [51,52]. They were developed to be chemically and functionally distinct from the other major classes of ergosterol-inhibiting antifungal drugs [10]. They act by interfering with early steps of ergosterol biosynthesis as a result of their specific, reversible, and non-competitive inhibition of the endoplasmic reticulum-associated enzyme squalene epoxidase, also called squalene monooxygenase, which is involved in the synthesis of sterols in fungi [52,53,54]. This enzyme, which is encoded by ERG1, together with (2,3)-oxidosqualene cyclase, is responsible for catalyzing a rate-limiting step of ergosterol biosynthesis in fungi by cyclization of squalene to lanosterol [51]. Squalene epoxidase uses the flavin adenosine dinucleotide (FAD) as a cofactor and obtains electrons from NADPH-cytochrome P450 reductase instead of binding NADPH directly in order to perform the epoxidation and the reduction of molecular oxygen to water [55]. Treated fungi would accumulate the sterol precursor squalene, while becoming deficient in ergosterol, an essential component of fungal cell membranes [56,57]. The resulting depletion of ergosterol and accumulation of squalene affect membrane structure and functions, such as nutrient uptake [58]. The fungicidal action of allylamines is closely associated with the development of high intracellular squalene concentrations that are believed to interfere with fungal membrane function and cell wall synthesis and to increase membrane permeability, thus leading to the disruption of cellular organization [59]. The ability of allylamines to accumulate more in the skin and nail beds compared to blood makes them highly effective antifungal agents against dermatophyte infections [60,61].

**Figure 3:**
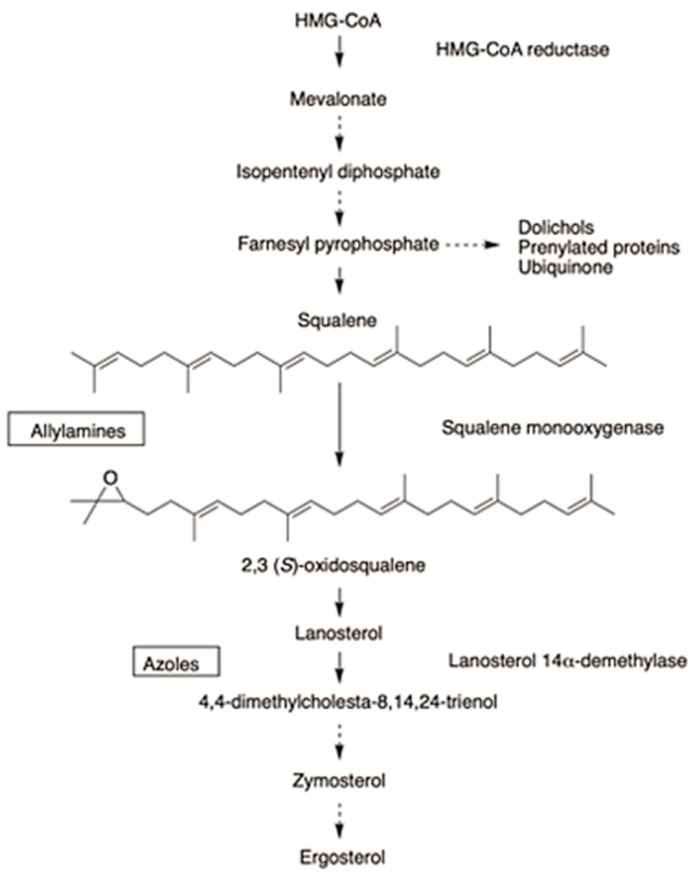
Ergosterol biosynthesis pathway in fungi [64]. The simple arrow indicates one catalytic step from the substrate to the product, and the dotted arrow represents the existence of several additional catalytic steps. The rectangular boxes show the site of action of allylamines and benzylamine on squalene epoxidase (squalene monooxygenase), which is distinct from the site of action of azoles.

The mechanism of action of the benzylamine, butenafine, is similar to that of allylamines, in addition to causing direct membrane effects in ergosterol-depleted fungal cells [51,62]. Iwatani et al. studied the mechanism of action of butenafine hydrochloride in *Candida albicans*, where the drug showed inhibition of squalene epoxidation, with 50% inhibitory concentrations of 0.57 microgram/mL, and induction of the release of appreciable amounts of inorganic phosphate (Pi) from *C. albicans* cells at 12.5 micrograms/mL. These findings suggested that the anticandidal activity of butenafine may be related to its direct membrane-damaging effect and the drug-induced alteration in the cellular sterol composition rendered the cell membrane more susceptible to damage [63]. The steps of ergosterol biosynthesis pathway and the inhibition by allylamines and benzylamines are shown in Figure 3.

## 4. Dosage Forms and Pharmaceutical Formulations of Allylamines and Benzylamines

### 4.1. Overview of Conventional Dosage Forms of Allylamines and Benzylamines

The allylamines, naftifine and terbinafine, as well as the benzylamine, butenafine, are available in various types of dosage forms detailed in Table 1. While naftifine and butenafine are available only as topical formulations, terbinafine is available as both topical formulations and as an oral tablet [65,66,67,68]. Topical delivery of antifungal drugs is perhaps the most effective route against cutaneous fungal infections for the various advantages they offer [69]. In fact, the topical route offers several advantages, including improved and site-specific drug delivery with reduced systemic adverse effects, enhanced patient compliance, in addition to avoidance of hepatic first-pass effect, and thus improved bioavailability and efficacy of treatment [65,69,70,71]. On the other hand, the conventional formulations have several drawbacks such as reduced drug penetration at the site of action, thus limiting local bioavailability and diminishing therapeutic efficacy, adverse skin reactions such as skin irritation, drug-induced hypersensitivity and allergic reactions, high dose and dosing frequency, leading to an increased risk of both local and systemic toxicity besides increased metabolism by local cytochrome P450 enzymes [72,73,74].

### 4.2. Novel Drug Delivery Systems of Allylamines and Benzylamines

For all the aforementioned reasons, novel drug delivery systems have emerged over recent years to overcome the problems associated with the conventional topical formulations of antifungal drugs [65]. In fact, formulation scientists have been developing novel nanopharmaceuticals-based drug delivery systems that have the potential to increase skin penetration, efficacy, and therapeutic potential while reducing toxicity [65]. Several nanoformulation strategies have been studied for delivering naftifine, terbinafine, and butenafine through targeted skin sites, such as microemulsions; vesicular carriers (including niosomes); and lipidic and polymeric particulate carrier systems [65].

#### 4.2.1. Microemulsions

Microemulsions are clear, isotropic, thermodynamically stable dispersions with a droplet diameter ranging between 10 and 100 nm, prepared using two immiscible liquids with the aid of a suitable surfactant [65]. They are an attractive formulation owing to their simplicity and lower cost, plus the enhanced cutaneous delivery and retention for a wide range of drug molecules including both hydrophilic and lipophilic drugs [65,75].

Microemulsion formulations of naftifine hydrochloride have been optimized and the efficacy of the formulation has been investigated where it was shown to have an enhanced permeation, thus localization of naftifine hydrochloride in the skin’s deeper layers [76]. Furthermore, butenafine hydrochloride has also been loaded in developed gelatin-containing microemulsion-based organogels, which showed its potential to be formulated as a transdermal drug delivery vehicle [77].

#### 4.2.2. Niosomes

Niosomes are a kind of liposomes prepared with nonionic surfactants [78]. After their topical application, they interact with the stratum corneum leading to a reduction in transepidermal water loss [79]. The skin permeation of niosomes depends on multiple factors including the types of surfactants, nature of drug, interaction between niosome and skin, and the composition, as well as the morphological characteristics of niosomal preparations [80,81]. Niosomes have several advantages over liposomes in terms of lower cost, increased skin permeation and higher chemical stability, leading to an increase in the product’s shelf life and the unique amphiphilic properties they possess that make them a suitable option for a wide range of drugs [69].

The development of an alcohol-free niosome gel containing naftifine hydrochloride and its optimization to achieve maximum physical drug stability and drug loading has been studied [82]. Moreover, terbinafine hydrochloride niosomes showed efficacy against *Aspergillus niger*, where in vitro findings showed that by increasing surfactant concentration the entrapment efficiency increases [83].

#### 4.2.3. Nanoemulsions

Nanoemulsions are colloidal dispersions of droplets with a size range of less than 1 µm (typically in the range 20–200 nm); they are either oil in water (o/w) or water in oil (w/o) dispersions, stabilized by an interfacial film of surfactant and co-surfactant [65]. Topical nanoemulsions offer many advantages including transparency, high stability, increased interfacial area, enhanced drugs’ skin penetration, and improved drugs’ solubility and, thus, bioavailability [65,84]. The literature has widely evaluated the optimization and characterization of topical nanoemulsions of various antifungal drugs [65]. Karri and co-workers prepared terbinafine hydrochloride loaded nanoemulsion, where their results revealed an increased skin permeation and thus better cure rates in animal models by overcoming the permeability and efficacy problems of the poorly soluble terbinafine hydrochloride [85].

#### 4.2.4. Dendrimers

Dendrimers are a specific class of polymers ranging between 10 and 100 nm in diameter and are widely used for drug delivery and imaging applications [65].

Khairnar et al. investigated the potential of polyamidoamine (PAMAM) and polypropyleneimine (PPI) dendrimers as tools for enhancing the antifungal activity of terbinafine, where in vitro findings demonstrated that the complexation of terbinafine with dendrimers lead to excellent antifungal activity compared to pure drugs themselves [86].

#### 4.2.5. Lipidic Nanoparticles

The first generation of lipid-based nanoparticles (NPs) is solid lipid NPs (SLNs), which are colloidal lipid carriers with a particle size ranging between 50 and 1000 nm. They are usually present in solid form at room and body temperatures and are capable of incorporating both hydrophilic and lipophilic drugs [65,87].

SLNs have been successfully used as an alternative to liposomes, lipid emulsions, polymeric NPs, and micelles for the various advantages they offer. These include high biocompatibility and biodegradability, drug stability against chemical degradation, flexible and controlled release, enhanced skin penetration and retention, increased therapeutic efficacy, reduced toxicity, as well as ease of scale-up and manufacturing [65,73,87,88,89].

On the other hand, the nanostructured lipid carriers (NLCs), the second generation of NPs, were designed to overcome the drawbacks associated with SLNs such as limited drug loading, gelation risk, and drug leakage during storage due to lipid polymorphism, where they are prepared by combining solid and liquid lipids [65,73,90]. Both SLNs and NLCs are attractive options where they have been investigated as suitable carrier systems to adjust drug delivery across various skin strata [65,91].

SLNs have gained interest for the topical treatment of cutaneous fungal infections. Vaghasiya et al. developed terbinafine hydrochloride loaded SLN where the ex vivo and in vivo studies showed that SLNs-based gel led to a higher skin retention, reduced systemic transport, decreased fungal burden and, thus, improved antifungal efficacy [92]. Another investigation showed that the application of terbinafine loaded SLN could reduce the administration period [93], whereas terbinafine loaded NLC showed a sustained release pattern and reduced fungal burden in a shorter duration of time [94]. The major properties of allylamines and benzylamines novel drug delivery systems are summarized in Table 2.

### 4.3. Penetration-Enhancing Strategies: Electroporation

To improve drugs’ permeability, various penetration-enhancing strategies can be employed such as electroporation [69]. Electroporation is a biophysical phenomenon that improves the drugs’ transdermal permeation by applying intermittent electric pulses, which change the cell membrane’s permeability transiently [69].

Novickij and co-workers investigated the skin permeation effects of pulsed electric fields with naftifine and terbinafine, where the results revealed increased sensitivity to drugs and higher inactivation of *C. albicans* [95].

## 5. Clinical Importance of Allylamines and Benzylamines

Allylamines and benzylamines remain the agents of choice for superficial dermatophyte infections. According to the results of a meta-analysis that pooled data from 65 clinical trials, there were no statistically significant differences among naftifine, terbinafine, and butenafine regarding the outcome of mycologic cure at the end of treatment. Butenafine hydrochloride and terbinafine hydrochloride were significantly more efficacious than clotrimazole, oxiconazole nitrate, and sertaconazole nitrate. Terbinafine also demonstrated statistical superiority when compared with ciclopirox, and naftifine hydrochloride showed better response compared with oxiconazole, justifying higher effectiveness than azole antifungals [96]. Furthermore, no differences were visible in safety nor tolerability [97], indicating these drugs as superior compounds to other antifungals in cutaneous dermatophyte infections, but with no consistent differences among each other. A presentation of clinical uses, spectrum, and main findings from trials carried out on the three antifungal agents is discussed below.

### 5.1. Naftifine

Formulated as a hydrochloride salt for topical administration in the form of 1% cream or gel, and 2% cream, naftifine is an allylamine derivative approved by the United States Food and Drug Administration (US FDA). The 1% preparations are indicated for twice-daily topical application for the treatment of tinea pedis, tinea cruris, and tinea corporis caused by *Trichophyton rubrum, T. mentagrophytes, T. tonsurans,* and *Epidermophyton floccosum*, for a duration 3–4 weeks. Naftifine 2% cream is FDA-approved for once-daily treatment of interdigital tinea pedis, tinea cruris, and tinea corporis caused by *T. rubrum* in adult patients for a duration of 2 weeks [98,99]. The majority of clinical data gathered on topical naftifine examined the 1% formulations once or twice daily, with once-daily administration generally being similar to twice-daily application in terms of effectiveness for cutaneous dermatophyte infections, with therapeutic success after a 2- to 5-week course in over 80% of patients with tinea cruris or corporis, and in a slightly smaller percentage of those with tinea pedis [100,101]. The high cure outcomes with naftifine may be explained by its lipophilicity, keratinophilic properties, fungicidal activity, and persistence of drug levels within the skin layers following discontinuation of topical application [96].

Naftifine exhibits in vitro fungicidal activity against a broad spectrum of dermatophytes, including *T. rubrum, T. mentagrophytes, T. tonsurans, E. floccosum, Microsporum canis, M. audouini, and M. gypseum* [98]. Early studies on naftifine have also shown its capacity to reduce growth and sterol biosynthesis in the opportunistic fungus *C. albicans* [102], as well as against *Asperillus* [101,103], although it is not approved for these uses. Naftifine demonstrated antifungal activity against the opportunistic yeast *Rhodotorula mucilaginosa*, which was isolated from an immunocompromised patient with onychomycosis, where the drug reduced biosynthesis of both ergosterol and carotenoid pigments, producing depigmented cells with modified structures [104]. Gold and colleagues [105] evaluated the efficacy and safety of naftifine 1% gel applied twice daily for 2 weeks in adults with tinea versicolor, a superficial fungal infection characterized by cutaneous pigmentary changes, itching, scaling, and erythema. Patients in this pilot study demonstrated improvement in symptoms without treatment-related adverse effects. Similar positive outcomes were obtained in another pilot study on patients with moderate seborrheic dermatitis of the scalp [106], perhaps highlighting the potential role of naftifine in various skin fungal infections. Interestingly, and while naftifine is not approved for treatment of bacterial infections, recent research on methicillin-resistant *Staphylococcus aureus* (MRSA) has shown that naftifine increases the susceptibility of this organism to photodynamic antimicrobial therapy by inhibiting the synthesis of the virulence factor staphyloxanthin [107]. The effect was also observed in mouse models at nanomolar concentrations of naftifine [108], providing insights for synergistic antibacterial effect on MRSA, and a rapid, efficient treatment for this multi-resistant bacterium. Apart from antimicrobial activities, naftifine demonstrated anti-inflammatory activity comparable to hydrocortisone [109]. The mechanism of such activity is thought to arise via a reduction in superoxide production and a reduction in polymorphonuclear leukocyte chemotaxis and endothelial adhesion [101].

In terms of safety, naftifine exhibits good local tolerability and absence of systemic adverse effects [100], due to poor systemic absorption [101]. During clinical trials with naftifine 1% cream, the incidence of adverse reactions was burning/stinging (6%), dryness (3%), skin tenderness (3%), erythema (2%), itching (2%), and local irritation (2%). Application site reactions such as burning, stinging, and itching are relatively uncommon, and reported in 2% of naftifine-treated patients compared with 5% for topical clotrimazole [98]. Minimal side effects were also reported in children treated effectively for tinea corporis by naftifine [110]. Allergic contact dermatitis has been reported with topical naftifine, with sensitization risk estimated at 1:100,000 [100,111]. Naftifine is pregnancy category B, but its safety in nursing women has not been established [112].

### 5.2. Terbinafine

This allylamine antifungal has been in clinical use for almost three decades and is currently approved as the gold standard treatment for oral use in onychomycosis, a fungal infection of the nail unit. About 90% of toenail and 75% of fingernail onychomycosis are caused by dermatophytes, notably *T. mentagrophytes* and *T. rubrum.* Clinical manifestations include nail discoloration, subungual hyperkeratosis, onycholysis, and onychauxis [113]. Although onychomycosis may be painful, patients usually present to dermatology clinics for cosmetic concerns associated with nail appearance. Treatment, especially in older adults and diabetic patients, is important, as onychomycosis can lead to cellulitis and foot ulcers in such populations, and is preferably given by the oral route [114]. A standard single daily dose of 250 mg of terbinafine is given orally for 6 weeks in fingernail onychomycosis and for 12 weeks in toenail onychomycosis in adults. Oral administration is also approved for tinea capitis in children aged 4 years and above [113,115]. In 2017, a Cochrane review was published to compare oral terbinafine to other antifungal medications in onychomycosis. The review evaluated 48 randomized controlled trials involving 10,200 participants, and evaluating terbinafine, griseofulvin, and azoles. Terbinafine was found to be effective for treatment compared with placebo, more effective than azoles for clinical cure, and with the same rate of adverse events as azoles. On the other hand, terbinafine was more effective than griseofulvin and with a lower rate of adverse events [116]. According to Gupta and Colleagues [117], in a meta-analysis of onychomycosis treatments, terbinafine 250 mg was significantly superior to all treatment regimens except itraconazole 400 mg pulse therapy. In a randomized, double-blind, controlled trial, the standard dosing of terbinafine was equally effective to pulse dosing, which includes three pulses of terbinafine of 500 mg each daily for a week, repeated every 4 weeks, regarding clinical and mycological cure rates [118]. However, the results of the meta-analysis are in favor of the classical continuous regimen for total mycological cure [119]. Compared to azole antifungals, a 1-week application of terbinafine 1% cream eradicated fungal pathogens in tinea pedis at least as effectively as 4-week courses with topical azoles and exhibited lower relapse rates. The high efficacy of short-term treatment with terbinafine in patients with tinea pedis may be related to its fungicidal activity in addition to drug reservoir formation in the upper layers of the epidermis [120].

Besides oral use in onychomycosis, topically, terbinafine 1% creams, solutions, and sprays are approved for tinea pedis, tinea corporis and tinea cruris in adults, for a duration ranging between one and four weeks [121,122]. In tinea pedis, a recent systematic review of randomized controlled trials indicated terbinafine (as well as butenafine, discussed shortly) as a most efficacious treatment [123]. Likewise, favorable significant clinical cure rates were reported with terbinafine for tinea cruris and tinea corporis according to the Cochrane review of clinical studies [124]. In a study from France [125] and another from China [126], the single application of a novel 1 % terbinafine film-forming solution was effective and well tolerated in the management of tinea pedis. In refractory types of tinea pedis, tinea corporis, and tinea cruris, oral terbinafine therapy may be recommended [127].

The clinical utility of terbinafine arises from its broad spectrum of antifungal activity against fungal dermatological infections. Primarily, terbinafine is fungicidal against dermatophytes, while a fungistatic activity is seen against *C. albicans* [128]. The minimum inhibitory concentration (MIC) of terbinafine versus dermatophytes such as *Trichophyton*, *Microsporum*, and *Epidermophyton* spp. ranges between 0.001 and 0.05 µg/mL and is therefore more potent than azole derivatives, for which reported MIC values range from 0.1 to greater than 10 µg/mL. Generally, the in vitro activity of terbinafine against dermatophytes exceeds that of other antifungal agents [129]. Nevertheless, the spectrum of terbinafine extends well beyond its use in acute and chronic dermatophytoses to include a wide range of subcutaneous and systemic mycoses [130]. In vitro, terbinafine is highly active against a broad spectrum of pathogenic fungi that cause cutaneous and lymphocutaneous sporotrichosis, aspergillosis, chromoblastomycosis, and other mycoses [131,132], although the results are somehow controversial [133,134,135]. Interestingly, in 2021, oral daily treatment with 500 mg of terbinafine in an elderly patient with chromoblastomycosis was synergistic with surgical debulking and intralesional amphotericin B, without reported adverse drug events during the course of treatment, and no recurrence after 6 months [136].

Examples of some of the side effects of oral terbinafine include headache, dermatitis, gastrointestinal distress, tiredness, malaise, taste disturbances, and liver enzyme abnormalities. However, according to expert clinical opinion [137], the risk of terbinafine-induced hepatotoxicity in healthy patients is considered negligible. Rare serious drug eruptions such as Stevens-Johnson syndrome may occur. Severe hepatic toxicity and thrombotic microangiopathy (including thrombotic thrombocytopenic purpura and hemolytic uremic syndrome) are rare potentially fatal side effects. Terbinafine is an inhibitor of CYP2D6, so clinicians should be alert of the potential for drug–drug interactions [138]. There are only minor adverse effects associated with the topical application of terbinafine, including local irritation, erythema, burning, rash and dryness, and its penetration into the systemic circulation is minimal, with not more than 5% of the topically applied dose being absorbed [127]. According to the results of a comparative study among pregnant women exposed to oral or topical terbinafine, no increased risk of major malformations or spontaneous abortion were identified [139]. If antifungal treatment cannot be delayed until after pregnancy, topical terbinafine when appropriate may be considered. Following oral administration, terbinafine is present in breast milk, while systemic absorption is limited following topical application [138].

As antimicrobial resistance is an inevitable evolutionary process in the microbial world, the emergence of resistance to antifungal therapy among dermatophytes is expected, and *T. rubrum*, *T. mentagrophytes* and *T. interdigitale* resistant to terbinafine have been documented [140]. In 2003, Mukherjee and Colleagues [141] published the first confirmed report of terbinafine resistance in dermatophytes in Cleveland, Ohio. Resistance was observed in six clinical *T. rubrum* isolates sequentially obtained from an onychomycosis patient recalcitrant to oral terbinafine therapy. Although normally susceptible to itraconazole, fluconazole, and griseofulvin, the isolates were fully cross resistant to several other squalene epoxidase inhibitors, including naftifine, butenafine, tolnaftate, and tolciclate, suggesting that such resistance was target specific. It is reported that mutations of squalene epoxidase result in structural changes which render terbinafine inefficient against this target; however, such structural changes do not have an effect on enzyme function [142], keeping it prevalent to sufficiently participate in the biosynthesis of sterols in the fungal membrane. It was suggested later that amino acid substitutions are likely to be responsible for terbinafine resistance in *T. rubrum* [143]. The Indian subcontinent was regarded as the initial niche where the original observations of high-level resistance in dermatophytes towards terbinafine were observed [144,145,146], with rates as high as 32% reported in one study [147]. However, a current epidemic including the spread of terbinafine-resistant dermatophytes is observed in many countries including France [148], Switzerland [149], and Japan [150], among others, and have been meticulously reviewed elsewhere [151]. In order to uncover mutations linked to terbinafine resistance or other mutated targets, Whole Genome Sequencing (WGS) can be a useful method. Dermatologists must be informed about terbinafine-resistant dermatophytes, and efficient systems must be implemented to identify them and survey their evolution.

### 5.3. Butenafine

Butenafine hydrochloride, a benzylamine derivative, as 1% cream, was first approved in Japan in 1992 for the treatment of tinea pedis, tinea cruris, tinea corporis, tinea versicolor, and superficial candidal infections. Clinical trials conducted in Japan demonstrated high efficacy rates and a low incidence of adverse effects, then the drug was approved in the US in 1997 for individuals aged 12 years and above [152]. The recommended therapy duration is 7 days, where it is applied twice daily in tinea pedis and once daily in tinea cruris and tinea corporis. In tinea versicolor, butenafine should be applied twice daily for 1 week or once daily for 2 weeks. In a multicentric, randomized, single-blind non-comparative study, which involved application of butenafine 1% cream in tinea pedis, tinea cruris, and tinea corporis, butenafine caused rapid resolution of signs and symptoms, including erythema, itching, burning, crusting, and scaling, with good patient and physician acceptance of treatment [153]. Furthermore, in another double-blind trial, butenafine caused higher clinical cure compared with clotrimazole at the end of 1 week in patients with clinically and diagnostically confirmed tinea cruris or tinea corporis [154]. The effectiveness of butenafine persists for at least 4 weeks following the discontinuation of therapy, suggesting its retention in the skin following termination of treatment [155]. This may be explained by the fact that butenafine readily interacts with membrane phospholipids of cutaneous tissues, allowing them to act as a local depot for the slow release of the drug, resulting in efficacious antifungal activity and long duration of action [156].

The chemical structure of butenafine is related to the allylamine class of antifungals with the exception that a butylbenzyl group replaces the allylamine group. Such structural alteration is claimed to relax spatial strain on the molecule, probably contributing to better antimycotic activity than naftifine and terbinafine [157]. Although clinical use of butenafine is restricted to the aforementioned tinea types, it has a broad spectrum of activity against dermatophytes, aspergilli, dimorphic fungi, and dematiaceous fungi [158,159]. In a study describing chemical synthesis of butenafine and its analogues and evaluation of their biological activity, butenafine was effective in vitro against the filamentous fungi *T. rubrum* and *M. gypseum*, as well as against the yeasts *Cryptococcus neoformans* and *C. gattii*. A demethylated analogue of butenafine, and its corresponding hydrochloride salt, was prepared by a short and simple synthetic route, and showed inhibitory activity against filamentous fungi, with reduced the burning sensation reported as one side effect of butenafine [160], anticipating its desirable effect as a promising product.

Besides the antifungal effect of butenafine, leishmanicidal effect has been demonstrated as well [161]. Leishmaniasis is a somehow neglected tropical and subtropical disease caused by an intracellular parasite from the *Leishmania* genus, transmitted to humans by the bite of a sand fly. Leishmaniasis is classified by the World Health Organization (WHO) among one of the seven most significant tropical diseases, and it represents a serious public health problem with a broad spectrum of clinical manifestations and a potentially fatal outcome. It is found on all continents except Oceania, and is endemic in some areas in Northeastern Africa, Southern Europe, the Middle East, Southeastern Mexico, and Central and South America [162]. Butenafine inhibits squalene epoxidase and suppresses the biosynthesis of ergosterol, an essential lipid of both fungal and leishmanial cell membranes [163], disrupting leishmanial membrane homeostais [164]; hence, it carries the potential of being repurposed for use in leishmaniasis. However, butenafine has limited oral bioavailability, with 1.5–3% of the oral dose being recovered in the plasma an hour after a single oral dosing of radiolabeled butenafine (0.2 mg/kg) [161], and is highly metabolized in the liver with only 0.03% of the oral dose recovered intact from the plasma after 4 h [68]. Butenafine self-nanoemulsifying drug delivery systems [161] and nanogels [165] were effective in animal models against cutaneous leishmaniasis. Furthermore, advanced oral solid nanomedicines enable non-invasive, safe administration of butenafine as a cost-effective and readily accessible repurposed drug for visceral leishmaniasis [161].

The common adverse effects associated with topical butenafine are burning, stinging, irritation, redness, and rarely contact dermatitis. Butenafine is pregnancy category C, and should be used with caution in breastfeeding mothers, as its excretion in human milk is unknown [166].

## 6. Conclusions and Future Directions

With potent and selective inhibition of squalene epoxidase, broad antifungal activity, significant accumulation in skin layers, and direct impact on fungal cell membranes, allylamines and benzylamines remain agents of choice for several fungal infections of the skin. The favorable potential of these compounds lies not only in their approved uses, but also in their anticipated effects on various other infection types, making them a possible anti-infective addition that warrants further investigation. With the current growing epidemic of chronic and/or recurrent fungal infections, and also the rise in resistance among dermatophytes, advances in formulation technology of these antimycotic agents are underway and promise to tackle such threats.

## Figures and Tables

**Table 1:** Conventional dosage forms of allylamines and benzylamines.

Antifungal Agent	Dosage Forms	Reference
**Allylamines**		
Naftifine	Cream (1–2%), gel (1–2%)	[65,66]
Terbinafine	Tablet (250 mg), solution (1%), cream (1%), spray (1%), gel (1%)	[65,67,68]
**Benzylamines**		
Butenafine	Cream (1%)	[65,66]

**Table 2:** Novel drug delivery systems of allylamines and benzylamines.

Drug Delivery System		Characteristics	Advantages	Studied on	Reference
Microemulsions		Clear, isotropic, thermodynamically stable dispersions Droplet diameter between 10–100 nm	Simple, lower cost, enhanced cutaneous delivery and retention Applicable for a wide range of hydrophilic and lipophilic drugs	Naftifine hydrochloride Butenafine hydrochloride	[65,75,76,77]
Niosomes		Liposomes prepared with nonionic surfactants	In comparison to liposomes: lower cost, increased skin permeation, higher chemical stability, increased product’s shelf life Suitable for a wide range of drugs	Naftifine hydrochloride Terbinafine hydrochloride	[69,78,82,83]
Nanoemulsions		Colloidal dispersions Droplets’ size of less than 1 µm (typically between 20–200 nm)	Transparent, high stability, increased interfacial area, enhanced drugs’ skin penetration, improved drugs’ solubility and bioavailability	Terbinafine hydrochloride	[65,84,85]
Dendrimers		Polymers between 10 and 100 nm in diameter	Widely used for drug delivery and imaging applications	Terbinafine	[65,86]
Lipidic Nanoparticles	Solid lipid NPs (SLNs)	First generation of lipid-based nanoparticles (NPs) Colloidal lipid carriers Particle size between 50 to 1000 nm	High biocompatibility and biodegradability, drug stability against chemical degradation, flexible and controlled release, enhanced skin penetration and retention, increased therapeutic efficacy, reduced toxicity, ease of scale-up and manufacturing Capable of incorporating hydrophilic and lipophilic drugs	Terbinafine hydrochloride	[65,73,87,88,89,92,93]
	Nanostructured lipid carriers (NLCs)	Second generation of NPs	Overcome SLNs’ drawbacks: limited drug loading, gelation risk, drug leakage during storage	Terbinafine	[65,73,90,94]

## Data Availability

Not applicable.
